# Will the promise of translational neuropsychopharmacology research ever deliver? The lion’s roar; the kitten’s purr

**DOI:** 10.1038/s44277-024-00005-w

**Published:** 2024-04-03

**Authors:** Jacqueline F. McGinty, Victoria Arango, Kathleen T. Brady, Sandra D. Comer, Rita Z. Goldstein, Eric J. Nestler, William W. Stoops, Michael A. Nader

**Affiliations:** 1 Department of Neuroscience, Medical University of South Carolina, Charleston, SC 29425 USA; 2 Adult Pathophysiology and Biological Interventions Development Branch, Division of Translational Research, NIMH/NIH Bethesda, Bethesda, MD 20892 USA; 3 Department of Psychiatry & Behavioral Sciences, Medical University of South Carolina, Charleston, SC 29425 USA; 4 New York State Psychiatric Institute and Columbia University Irving Medical Center, New York, NY 10032 USA; 5 Departments of Psychiatry & Neuroscience, Icahn School of Medicine at Mount Sinai, New York, NY 10029 USA; 6 Nash Family Department of Neuroscience and Friedman Brain Institute, Icahn School of Medicine at Mount Sinai, New York, NY 10029 USA; 7 Departments of Behavioral Science, Psychiatry and Psychology, Center on Drug and Alcohol Research, University of Kentucky, Lexington, KY 40506 USA; 8 Department of Translational Neuroscience, Wake Forest University School of Medicine, Winston-, Salem, NC 27157 USA

**Keywords:** Translational research, Diseases of the nervous system

## Abstract

The gap between neuropsychopharmacology research claims (*the lion’s roar*) and effective treatments for neuropsychiatric disorders (*the kitten’s purr*) persists. However, a pattern of purrs over time may be as important as a loud roar. This perspective pulls together diverse preclinical and clinical voices of major figures in the neuropsychopharmacology research field to address how inter-disciplinary scientific approaches progress from thinking about the brain and its disorders to testing novel hypotheses to implementing treatments that may improve brain health in individuals with neuropsychiatric disorders.

## Lay Summary

This perspective probes the advantages and disadvantages of basic scientific and clinical research into brain health and neuropsychiatric disorders and how investigators in each discipline may more faithfully translate their discoveries into treatments for brain disorders.

## Introduction

**Fig. 1:**
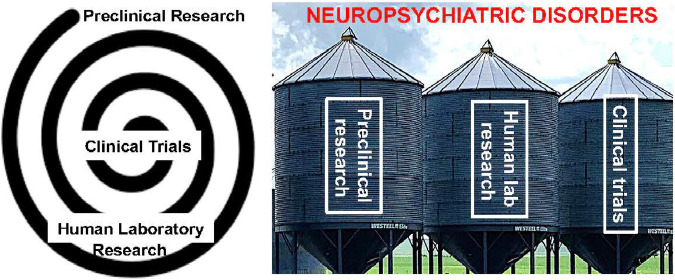
Ideally neuropsychopharmacological research would flow bidirectional to/from animal and human studies all the way to clinical trials of promising therapeutics (left). More often the process exists in silos performed by independent teams without sufficient bridging between the different needed steps and phases (right).

The process of discovery and testing hypotheses in brain research into neuropsychiatric disorders classically began following one of two paths. In one, animal models were used to identify and modulate a promising therapeutic target, then those findings were translated into human laboratory studies, culminating in clinical treatment trials. In the other, neuropsychological, and later neuroimaging, tools were used in the human laboratory (or at bedside), with animal and cell models then used to understand the underlying mechanisms. Regardless of the starting point, however, these studies are commonly carried out in silos by independent investigators who exclusively use one or the other (animal vs. human) approach, creating a discontinuous, broken chain (Fig. 1). Specifically, too often, preclinical investigators do not advocate for their published discoveries to be translated into human lab studies and/or clinical treatment trials. In parallel, clinical researchers may not back-translate their research findings from human subjects to animal models. Together, these roadblocks inhibit the mechanistic understanding of core human neuropsychiatric disorders.

## Advantages and disadvantages of animal and human studies

There are several advantages to using animals to understand human disease processes. One advantage of animal research is that these studies can start with individual animals before a disease process begins by taking measurements, for example, in the case of substance use disorders (SUDs), in drug-naïve animals and testing their vulnerability to drug exposure. An emerging goal for these researchers is to discover (a) individual differences in vulnerability and (b) why a therapeutic approach works well in some animals and not in others: that is, to identify individual-level and sex differences in therapeutic responsiveness, and to avoid looking for a “magic bullet” that will effectively treat all subjects equally. Support for this point of view comes from PET imaging of G-protein-coupled receptors in nonhuman primate research in which receptor availability in key reward-related brain areas is altered differentially by addictive substances according to the sex and rank of socially housed monkeys [1, 2]. Importantly, the use of blood-based biomarkers can be studied and translated to the human condition [3]. The same strategies can be implemented for other neuropsychiatric diseases.

A disadvantage of animal studies is that they often provide only a snapshot of a particular phase of a pathophysiological process in highly controlled laboratory environments. That is, they are yet to consider strategies that address symptom and etiological heterogeneity or to adopt longitudinal observations within the same individual in more naturalistic settings. Therefore, because such designs do not allow for tracking of the time-changing/dynamic and complex nature of some of the underlying and/or predisposing mechanisms or to explore gradations and trajectories in the recovery process, the experimental designs of animal studies often are only remotely relevant to humans. Yet, closing this gap is possible as shown in, for example, treatment-resistant depression, in which a multi-scale systems biology approach with the goal of identifying biomarkers to facilitate the characterization of patient subpopulations, has emerged [4]. It must also be acknowledged that only a subset of human behaviors and behavioral disorders are amenable to study in nonhuman model systems [5].

Human laboratory studies can address some of these animal model limitations. One advantage of human lab studies is that they allow longitudinal within-subject designs, encouraging the study of different time points in both the disease process and its recovery. These studies are also amenable for exploring individual differences, embracing heterogeneity while adapting a whole-brain approach for purposes of circuit/network neuroscience. The opportunity to study behavior in naturalistic settings to enhance ecological validity, inclusive of clinical relevance and generalizability of results, has also been recently advocated, contributing to the performance of large-scale big-data science outside the confines of the lab. Such studies may advance the field by, for example, using neuroimaging and/or neuropsychological tools to identify non-invasive, easy-to-obtain and scalable biomarkers of brain function and structure. If translated back to animals, such biomarkers can be used to identify (causative) mechanisms underlying brain and behavioral pathology that can guide more selective medication/intervention development and testing.

A disadvantage of human lab studies is that the assignment of people to different psychopathological groups is outside the control of the scientist. Hence control over a myriad of related and unrelated factors is impossible (and/or would create non-representative groups), prohibiting the assignment of results to a clear-cut single variable. Given the common absence of a baseline study, i.e., conducted before disease/symptom onset, human lab studies also cannot address the eternal chicken-and-egg question (was the deficit there before disease onset, did it lead to disease development in the first place, or is it the cause of the disease). It is also, of course, not possible to intervene for research purposes physically in the brain to understand the function of individual genes, proteins, and pathways, which can be probed in preclinical studies. This makes studies in animals, and perhaps cell models, essential.

### Gaps in neuropsychiatric research

Despite the issues addressed above with both animal- and human-subjects research, many therapeutics for neuropsychiatric disorders have been approved by the FDA during the past decade. Some notable examples are brexanolone (intravenous infusion) and the related zuranolone (oral pill) approved for postpartum depression (PPD) [6]. These treatments, which are based directly on animal research involving neurosteroids performed 20–30 years ago [7], elevate women’s health issues by recognizing PPD as a real disorder previously suffered in silence by many women. Esketamine is another revolutionary example; it is a fast-acting antidepressant with fundamentally different underlying mechanisms of action than the decades-old, delayed-action drugs based on inhibiting serotonin or norepinephrine uptake [8]. For people suffering from opioid use disorder (OUD), several drugs that target the endogenous μ-opioid receptor system [9], such as buprenorphine, naltrexone, and methadone, have been approved. Moreover, neuromodulatory approaches such as transcranial magnetic stimulation, transcranial direct current stimulation, focused ultrasound, and deep-brain stimulation have a promising future for treating neuropsychiatric disorders [10, 11].

To decrease clinical trial failures, address long-standing barriers to existing treatment, and expedite progress, back translation from human to animal studies is crucial. For example, in the case of OUD treatment, the FDA-approved drugs were identified primarily in animal studies. However, they have been less effective in humans for many different reasons: species differences in drug metabolism, adverse effects that differ by species, and non-medical factors such as adherence to taking the medication as prescribed. Adherence is a problem, especially with drugs like naltrexone that block μ-opioid receptors and completely prevent the ability to “get high”. Further, the effect sizes of animal experiments are typically much smaller than what is required in human studies to predict treatment outcomes. Additionally, recognizing that comorbidities, polydrug intake, genetic, and sex differences – among other factors – influence treatment response will increase the effectiveness of therapies in and across subpopulations. To this end, stratifying participants into treatment arms based on biological subtypes such as those with different variations in mu opioid receptor genes (OPRM1), metabolic profiles for cytochrome P450 CYP2D6, dopamine D2 receptor levels, inflammatory biomarkers, or B cell activity could yield important information about how to more effectively treat a broader range of patients. Relatedly, using artificial intelligence to analyze multimodal big datasets will allow a better understanding of complicated trajectories and identification of personalized medicine approaches, accelerating progress in treating neuropsychiatric disorders. In this quest, new treatment endpoints must be identified and validated as clinically meaningful (e.g., one cannot expect therapies to promote complete abstinence in SUDs or effective responses to a particular therapy in 100% of patients with other neuropsychiatric disorders). Standardized and FDA-sanctioned use of non-standard, yet sensitive, outcome measures in research investigations could facilitate reproducibility across studies. An example is augmenting the common use of urine tests for drugs or length of abstinence with measures of quality of life and functioning, as well as separate assessments of “drug liking” and “drug wanting/craving/cue reactivity” in SUD research [12].

## Future directions

Disappointment in the progress of identifying and characterizing treatments for neuropsychiatric disorders over the last 40 years should be tempered by the realization that the neuroscience field is not sufficiently mature to succeed at a fast pace. First, only within the last decade have neuroscientists been able to phenotype single cell types across different brain areas and identify their function in specific circuits. For the first time, we now have the tools to identify the molecular contents of single cells, how they control their autonomous functions in a neural circuit, how that circuit is embedded in a network that creates behavior and how that behavior feeds back to alter the circuit as well as the molecular contents of the cells involved in an iterative process. Second, disappointing outcomes due to the lack of such mechanistic knowledge underlying select neuropsychopharmacological approaches have led to their premature deprioritization. Third, the concentration of investigators on a specific, funded research area gives rise to a certain complacency that, once a study is published, underlies a lack of working toward introducing new discoveries into the therapeutic pipeline. Strict research specialization hinders interdisciplinary communication and the ability to close the loop between translating preclinical and clinical studies into effective treatments.

The prolonged lag in advancing novel therapeutic targets into effective treatments for patients demonstrates that critical interactions between animal and human researchers and clinical trial investigators within academia and industry need to be improved. Therefore, we still need to find additional ways to close the gap between preclinical and clinical research to facilitate the identification and characterization of more novel treatments. One critical way is for preclinical and clinical researchers to work more closely together starting with establishing interdisciplinary teams to train young investigators (students, postdoctoral fellows, and medical residents) [13]. Educating basic scientists in the numerous social determinants (trauma, socioeconomic status, family dynamics, etc.) of behavioral health in humans that impact the wide heterogeneity of treatment responses is critically important. Further, educating clinical scientists about the rationale and experimental designs of animal studies and the neurobiology of neuropsychiatric disorders gleaned from these studies is similarly critical. These objectives can be accomplished best by creating collaborations that bring preclinical and clinical researchers together in regularly scheduled meetings across training programs [13]. It is also critical to educate the public about new promising treatment approaches, which is particularly relevant to neuromodulatory and future gene therapy approaches [10], as well as to understand whether communities will accept novel treatments. Introduction to new approaches to digital phenotyping via internet cognitive behavioral therapy (iCBT), smart phones and wearable devices such as smart watches hold promise in aiding diagnosis and treatment response/monitoring is imperative. Examples include iCBT for depression [14], remote clinical trials for recruitment and screening, assessment, biomarker collection, and medication adherence monitoring [15], and smartphone-enabled monitoring of breath carbon monoxide readings to determine smoking abstinence [16].

Moreover, we need to rekindle the practice of experimental human pharmacology. As just one example, the anti-anhedonia effects of a kappa opioid receptor (KOR) antagonist were identified 20+ years ago [17]. The underlying signalling mechanisms focused on the regulation of the endogenous opioid peptide, dynorphin – the natural ligand for KOR, by the transcription factor, cyclic AMP response element binding protein (CREB), in the nucleus accumbens of rodents. However, it took until 2020 for a placebo-controlled, double-blind study to be published demonstrating that a KOR antagonist decreased anhedonia, a major symptom of major depressive disorder, in a biomarker-based proof-of-mechanism clinical trial [18]. There are several reasons: it took time for a selective, high-affinity KOR antagonist with a favorable pharmacologic and safety profile and without major side effects to be developed and used as a PET ligand to provide biomarker evidence that it engaged KOR effectively at tolerated doses in humans. This study was the first comprehensive NIMH-sponsored Fast-Fail study that included a rigorous determination of whether a ligand is highly selective for its neurobiological and therapeutic target, reducing the vulnerability to bias and nonspecific effects in clinical trials. Despite the number of years that elapsed from discovery science to the clinical trial, this study is an excellent example of closing the past leaky pipeline of drugs tested in clinical trials by fulfilling evidence-based, rigorous neurobiological criteria that underlie positive behavioral outcomes in Phase 2 trials.

## Conclusions

Following several key principles will move translation in the field forward. First, for all models used, longitudinal, within-subject studies across time is important for testing interventions during different critical phases in disease progression. There is also an urgent need to characterize the main, non-linear and complex trajectories of recovery, for example by studying it in naturalistic settings to enhance ecological validity (e.g., by sampling select behaviors across different contexts). Together, such designs will facilitate identifying predisposing variables and biomarkers of the different phases of select pathophysiological processes and their treatments. Finally, employing bidirectional translation is a top priority. In the future, it will be critical to close the loop between preclinical and clinical studies (Fig. 1) by identifying new pathways and computational models to link animal-to-human translation and human-to-animal back-translated studies [19].
